# Variation of preventive practices and its association with malaria infection in eastern Indonesia: Findings from community-based survey

**DOI:** 10.1371/journal.pone.0232909

**Published:** 2020-05-07

**Authors:** Mara Ipa, Mutiara Widawati, Agung Dwi Laksono, Ina Kusrini, Pandji Wibawa Dhewantara

**Affiliations:** 1 Pangandaran Unit for Health Research and Development, National Institute of Health Research and Development, National Ministry of Health of Indonesia, Pangandaran, West Java, Indonesia; 2 Center of Research and Development of Humanities and Health Management, National Institute of Health Research and Development, National Ministry of Health of Indonesia, Jakarta, Indonesia; 3 Magelang Unit for Health Research and Development, National Institute of Health Research and Development, National Ministry of Health of Indonesia, Magelang, Central Java, Indonesia; Instituto Rene Rachou, BRAZIL

## Abstract

**Background:**

Geographical variation may likely influence the effectiveness of prevention efforts for malaria across Indonesia, in addition to factors at the individual level, household level, and contextual factors. This study aimed to describe preventive practices at individual and a household levels applied by rural communities in five provinces in eastern Indonesia and its association with the incidence of malaria among adult (≥15 years) populations.

**Methods:**

This study analyzed a subset of data of nationally representative community-based survey 2018 Riset Kesehatan Dasar (Riskesdas). Data for socio-demographic (age, gender, education and occupation) and preventive behaviors (use of mosquito bed nets while slept, insecticide-treated mosquito nets (ITNs), mosquito repellent, mosquito electric rackets, mosquito coil/electric anti-mosquito mats, and mosquito window screen) were collected. Data were analyzed using bivariate and multivariable logistic regression model.

**Results:**

Total of 56,159 respondents (n = 23,070 households) living in rural areas in Maluku (n = 8044), North Maluku (n = 7356), East Nusa Tenggara (n = 23,254), West Papua (n = 5759) and Papua (n = 11,746) were included in the study. In the multivariable models, using a bed net while slept likely reduced the odds of self-reported malaria among Maluku participants. Reduced odds ratios of self-reported malaria were identified in those participants who used ITNs (North Maluku, ENT, Papua), repellent (Maluku, West Papua, Papua), anti-mosquito racket (ENT), coil (Maluku, North Maluku, Papua) and window screen (West Papua, Papua).

**Conclusion:**

Our study concluded that the protective effects of preventive practices were varied among localities, suggesting the need for specific intervention programs.

## Introduction

Malaria is a significant communicable disease, and in 2018, this disease caused 405,000 deaths worldwide [1]. In 2018, an estimated US$ 2.7 billion was invested in malaria control and elimination efforts globally by governments of malaria-endemic countries and international partners. Of which, six percent of the total investments were spent In the South-East Asia region [1]. The world agreement on eliminating malaria is outlined in the WHO global target of elimination in 2030 with a milestone for measuring progress in 2020 and 2025 [2]. According to the World Health Organization, the incidence rate of malaria presents an 18% reduction between 2010 and 2017. Despite the significant reduction in the past decade, malaria incidence remains high.

Approximately 1.8 billion people in the Southeast Asia region, including Indonesia, remain at risk of getting malaria; it is more than half of the malaria global risk estimation [3].

Indonesia declared as one of the malaria-endemic countries [4]. At the end of 2018, there have been approximately 180 thousand confirmed malaria cases reported across Indonesia from 26 districts of malaria-endemic areas [5]. A considerable number of malaria cases were reported from Eastern Indonesia, including Papua, West Papua, East Nusa Tenggara, North Maluku, and Maluku. The Indonesian basic health research report says that the prevalence of malaria in Papua, West Papua, East Nusa Tenggara, North Maluku, and Maluku, respectively were 12.07%, 8.64%, 1.99%, 1.36%, and 1.21% [6].

To control malaria, the Indonesian government delivered some elimination programs through an integrated approach, particularly in endemic areas [7,8]. This integrated approach includes continuous distribution of insecticide-treated bed nets every two years in highly endemic districts, the distribution of Artemisinin combination therapy (ACT), and Insecticide Residual Spray [4,9]. Even though significant progress on this integrated malaria control has been made by the Indonesian government to reduce the burden of malaria, it remains an infectious disease of public health significance in Indonesia, affecting impoverished and remote rural communities. This situation could abort the fulfillment of Indonesia’s malaria elimination goal as well as the sustainable development goal.

In addition to factors at the individual level, household level, and contextual factors, geographical variation may likely influence the effectiveness of prevention efforts across Indonesia. Preventive measures of malaria varied between rural and urban communities [10,11], which could be resulted due to socioeconomic and development disparity between rural and urban [12]. Communities living in less developed areas or rural areas might have a lack of access to improved housing, essential health services, effective and timely diagnosis and treatment that might contribute to the higher risk of the transmission of malaria. Studies also have shown that financial problems have been a significant challenge in delivering malaria prevention and treatment program in rural communities [12–16].

Malaria transmission is associated with multiple factors, such as ecosystems and socio-ecological status. The ecosystem factors consist of Plasmodium as parasites, Anopheles mosquitoes, and human hosts. Socio-ecological status factors include gender, age, occupation, and behaviors. In addition to contextual factors, individual and household preventive practices can also affect the effectiveness of a malaria prevention effort. The previous research regarding malaria prevention documented varied factors associated with malaria prevalence existed at individual and community level. These factors include the characteristics of participants, lack of knowledge about the availability of healthcare services, and unimproved housing [7,17]. At household-level, malaria risk factors determined by the number of inhabitants, household condition, household economic condition (income), insecticide-treated nets (ITNs) ownerships, and the ability to access healthcare facilities [17–20]. Some research also shows prevention efforts at the individual level include the use of repellent, mosquitoes coil, electric rackets, and electric mosquito repellent [21–23]. In our knowledge, there is a shortage of evidence regarding variation in preventive practices applied by rural communities in eastern Indonesia.

This study aimed to describe preventive practices at individual and household levels applied by rural communities and its association with the incidence of malaria among adult populations in eastern Indonesia. The results of this study could be beneficial to assist local health authorities in designing better and implement prevention programs to reduce the risk of malaria in eastern Indonesia.

## Materials and methods

### Study sites

The present study was limited to five provinces in eastern Indonesia, including Maluku, North Maluku, East Nusa Tenggara (ENT), West Papua, and Papua province. Maluku province consists of 11 districts. It is an archipelago with more than 600 islands stretch between North Maluku and Ceram Sea (north), West Papua province and Arafura Sea (east), East Timor and Timor Sea (east) and the province of Southeast Sulawesi and Banda Sea (west). Maluku province has a population of 1.5 million people and an area of 54,185 km^2^ with elevation ranging from 3 to 3027 m [24]. Both West Papua and Papua provinces are situated in the west part of the Papua islands which border to Papua New Guinea in the east. West Papua province consisting of 12 districts and one municipality, inhabited by more than 800,000 people with an area of 99,671 km^2^ [25]. While Papua province, including 28 districts and one municipality, and it has a population of 2.8 million people with an area of 316,553 km^2^ [26]. Papua islands have elevation ranging from 1.17 m to more than 4000 m, with the highest altitude is found in the Puncak Jaya district. The ENT is known as *“Flobamorata*,*”* which comprises of five major islands: Flores, Sumba, Timor, Alor, and Lembata. The total land area is 47.931,54 km2, with Timor island as the largest island (14.732,35 km^2^). This islands comprises of 21 districts and a municipality, inhabited by approximately 4.6 million people [27]. North Maluku covers an area of 31,982.50 km^2^, and it consists of 8 districts and two municipalities. These islands are inhabited by 1,038,087 people [28].

### Study design and data collection

This study was a secondary analysis of the 2018 Indonesia Basic Health Research (Riset Kesehatan Dasar, RISKESDAS 2018). The 2018 RISKESDAS was a cross-sectional, quinquennial, and nationally representative, community-based survey. This survey aimed to assess public health indicators key for policymaking at national, provincial, and district-level. Those indicators include access and health services, environmental health, housing, and economy, communicable and non-communicable diseases, health financing, reproductive health, children’s health, and immunization. The survey was conducted by the National Institute of Health Research and Development (NIHRD) Ministry of Health of Indonesia in 34 provinces, held from April to May 2018. Household samples were chosen based on the stratified multistage systematic random sampling design and the probability proportional to size (PPS) method. At the first stage, 30,000 primary sampling units (PSUs) were chosen using the PPS technique based on the 2010 Indonesia Population Census. Each PSUs consisted of several census blocks (CBs), which defined as the enumeration areas (EA) of the census. The second stage was the selection of CBs at each selected PSU using PPS based on household numbers estimated from the 2010 Indonesia Population Census. The third stage was randomly selected 25 households from selected CBs. In total, the 2018 RISKESDAS surveyed 295,720 households, 1,091,528 household members in 34 provinces as a sample of 2018 RISKESDAS. The CB was excluded if it could not be accessed due to natural disasters, social unrest/conflict, or extreme geographic condition. RISKESDAS data collection was carried out by trained enumerators. Enumerators were instructed on how to use the questionnaire and on ways to approach respondents and obtain consent. These enumerators visited selected households accompanied by local health authorities and village leaders. Before the interview, consent forms were given to all members of the family. Parents or guardians (household heads, their spouses, or an older representative of the house) and all household members who consented to participate in the study were interviewed. A parent or guardian accompanied those respondents aged less than 15 years old during the interview. The enumerators collected data using a paper-based structured questionnaire. The questionnaire consisted of household-level and individual-level sections. Data were then entered into data entry software of the Census and Survey Processing System (CSPro) 7.3.

### Data

For this analysis, we analyzed a subset of data of RISKESDAS 2018. We restricted our analysis on samples aged ≥15 years for this study (N = 56,159) living in rural areas in five endemic malaria provinces: Papua (n = 11,746), West Papua (n = 5759), Maluku (n = 8044), North Maluku (n = 7356) and East Nusa Tenggara (n = 23,254). We chose this as it corresponds to the age group within the age range defined as adolescent in Indonesia [29]. It clarified that the age range of adolescent is mature in thought and capable of decision taking [30]. Additionally, these five provinces were chosen as these areas are hyperendemic and the main contributor (165217, n = 91.68%) on the national notified malaria cases in Indonesia (2018 Indonesia Health Profile) [31].

### Variables

In this analysis the outcome variable was self-reported malaria; a dichotomous variable coded as “1” if a member of the household was asked whether he / she has been diagnosed in the past 12 months by local health care providers / physicians as having positive laboratory confirmed malaria before the survey and “0” if he/she reported otherwise. The answer to this question was binary: code 1 (Yes) and code 0 (No). Malaria has typically been confirmed in health facilities using Rapid Diagnostic Tests (RDTs) and microscopy. For this study, no screening tests were carried out by the interviewer. We used “self-reported malaria” as outcome variable because the survey was not collected blood samples and did not use any diagnostic test to confirm malaria infection among respondents. This variable was based on respondents’ recall whether or not they have been laboratory diagnosed malaria in the past 12 months.

The explanatory variables were classified into two aspects: socio-demographic and preventive behavior. Socio-demographic variables were age (grouped into 2 categories: 15–24 and more than 24 years), gender (male/female), education (not school, completed primary, secondary or tertiary education), occupation (not working, farmer or non-farmer). Preventive practices variables were defined into individual level and household level. Respondents were asked whether he/she: a) used bed net while slept last night before the survey (Yes/No), used ITNs for ≤ 3 years (Yes/No), have used ITNs for > 3 years (Yes/No), often used mosquito repellent (Yes/No) and often used mosquito electric rackets (Yes/No). In this study we used both variables of whether people have used ITNs for less than 3 year or more. These variables were used in the survey as it is relevant with what WHO have advised [32]. These variables are associated with the durability of ITN to effectively prevent mosquito bites (efficacy). In addition, at household-level, respondents were asked whether he/she: a) often used mosquito coil anti-mosquito mats (Yes/No) and b) installed window screen (Yes/No) to prevent mosquito bites. In term of variable of the use of repellent and coils/mats, code 1 (“Yes”) was given if respondent routinely used repellent/coils/mats each day until the day before the survey.

### Statistical analysis

Descriptive analysis was employed to describe general characteristics (numbers, frequent, and proportions) of the explanatory variables. Bivariate regression analysis was conducted to test the associations between malaria and the explanatory variables. Variables significant at *p*-value <0.2 in the bivariate model were included in the multivariable logistic regression model. Multi-collinearity among explanatory variables was examined using the variance inflation factor (VIF) before recruiting variables to the final mode. Multivariable logistic regression analysis was done to identify factors associated with malaria after adjusting for potential confounders. In the final model, statistical levels of significance were evaluated at 5%. The odds ratios (ORs) and 95% confidence intervals (CIs) were reported. Due to the complex nature of the sampling structure of the 2018 RISKESDAS data, we applied complex data analysis. All statistical analyses were performed with SPSS 21 (Chicago, IL, USA). The map of the prevalence of malaria was generated in ArcGIS 10.5 ((ESRI Inc., Redlands, CA, USA). The shapefile of administrative boundary polygons were obtained from the Bureau of Statistics of Indonesia (Badan Pusat Statistik—Sistem Informasi Layanan Statistik) (http://www.silastik.bps.go.id).

### Ethics approval and consent to participate

The 2018 RISKESDAS protocol was reviewed and approved by The National Ethics Commission approved it for Health Research, National Institute of Health Research and Development (NIHRD), Ministry of Health of Indonesia (Number: LB.02.01/2/KE.024/2018). Respondents have provided written approval for their involvement in the study. For this analysis, the respondents' identities have all been removed from the dataset.

## Results

### Socio-demographics characteristic of participants

The socio-demographic characteristics of the participants were given in Table 1. A total of 56,159 respondents (23,070 households) resided in rural areas in five provinces were included in the analysis. In all provinces, most respondents included in the study were adults older than 24 years (76.4%), while the gender representation in the sample appeared to be balanced. In Maluku (51.3%), North Maluku (47%), and West Papua (48.8%), a large percentage of respondents had attained secondary level education. While, in Papua, most of the respondents were not achieved or not completed primary school (45.6%). In all provinces, a small number of respondents had tertiary degrees, ranged from 3.8–7.9%. Based on the type of occupation, the majority of respondents in Maluku (39%), East Nusa Tenggara (52.7%) and Papua (59.1%) were engaged in farming. Whereas, in North Maluku and West Papua, approximately one-third of respondents involved in non-agricultural and were not working, respectively.

**Table 1:** General characteristics of the respondent who included in the analysis, in the five selected provinces in eastern Indonesia.

	Maluku (N = 8044)		North Maluku (N = 7356)		East Nusa Tenggara (N = 23,254)		West Papua (N = 5759)		Papua (N = 11,746)		Total (N = 56,159)	
	n	%	n	%	n	%	n	%	n	%	n	%
**Age (years**)												
15–24	1831	25.6	1583	24.3	4781	25.2	1258	24.1	2281	19.9	11734	23.6
25 and above	6213	74.4	5773	75.7	18473	74.8	4501	75.9	9465	80.1	44425	76.4
**Gender**												
Male	3773	50.1	3509	50.6	10919	48.4	2829	53.0	5943	52.0	26973	50.1
Female	4271	49.9	3847	49.4	12335	51.6	2930	47.0	5803	48.0	29186	49.9
**Education**												
No education	1238	12.9	1373	16.7	6900	27.1	1282	17.9	4981	45.6	15774	29.0
Primary	2543	27.9	2168	28.4	7919	33.0	1308	20.8	2288	18.1	16226	27.1
Secondary	3696	51.3	3252	47.0	7106	34.0	2558	48.8	3956	32.5	20568	37.8
Tertiary	567	7.9	563	7.9	1329	6.0	611	12.5	521	3.8	3591	6.2
**Occupation**												
Not working	2873	36.8	2217	28.7	6310	28.3	2211	37.8	3471	26.9	17082	29.5
Farmer	3588	39.0	3182	28.5	12951	52.7	1801	26.8	6478	59.1	28000	50.3
Non-farmer	1583	24.2	1957	42.8	3993	19.0	1747	35.4	1797	14.0	11077	20.2
**Individual preventive measure**												
Used mosquito nets while slept last night	1159	13.5	1224	16.0	4396	20.3	968	16.3	3022	22.4	10769	19.5
Used insecticide-treated mosquito nets ≤ 3 years	4206	43.3	3230	41.9	14842	61.8	3380	54.2	5182	39.7	30840	51.2
Used insecticide-treated mosquito nets > 3 years	1282	15.9	1043	13.7	4725	19.0	1093	14.7	2172	13.7	10315	16.4
Used mosquito repellent	1215	17.4	2385	32.2	1758	9.5	1229	25.4	1555	12.8	8142	14.5
Used mosquito electric rackets	196	3.0	325	5.0	594	2.6	287	6.8	271	2.1	1673	3.0
**Household-level preventive measure**												
Used mosquito coil/electric anti–mosquito mats	2524	35.9	3567	50.8	3356	17.1	2029	37.5	2885	22.9	14361	25.4
Installed anti-mosquito window screen	374	3.5	310	4.3	675	3.2	887	19.6	1457	11.2	3703	6.6

Approximately twenty percent of the total respondents used the bed net while sleeping. About 20% of respondents in East Nusa Tenggara and Papua reported that they used a bed net when they slept. While in other locations, less than 20% of respondents used the bed net. Half of the total respondents indicated that they utilized ITNs for less than five years. Among those five provinces studied, the largest proportion of respondents that used ITNs less than five years was found in East Nusa Tenggara (62%). No more than 20% of respondents used ITNs for more than five years in all provinces. About one-fourth of the West Papuan respondents reported that they used repellent. While there were few respondents in East Nusa Tenggara used repellent (9.5%). Few respondents said that they used electric racket to prevent malaria.

At the household-level, approximately 17% (East Nusa Tenggara) to 50% (North Maluku) of the respondents burnt mosquito coil or used electric anti-mosquito mats to prevent malaria. Respondents in West Papua (19.6%) and Papua (11%) reported that they used mosquito window screen. No more than 5% of respondents in Maluku, North Maluku and East Nusa Tenggara used window screen.

### Self-reported prevalence by districts

**Fig 1:**
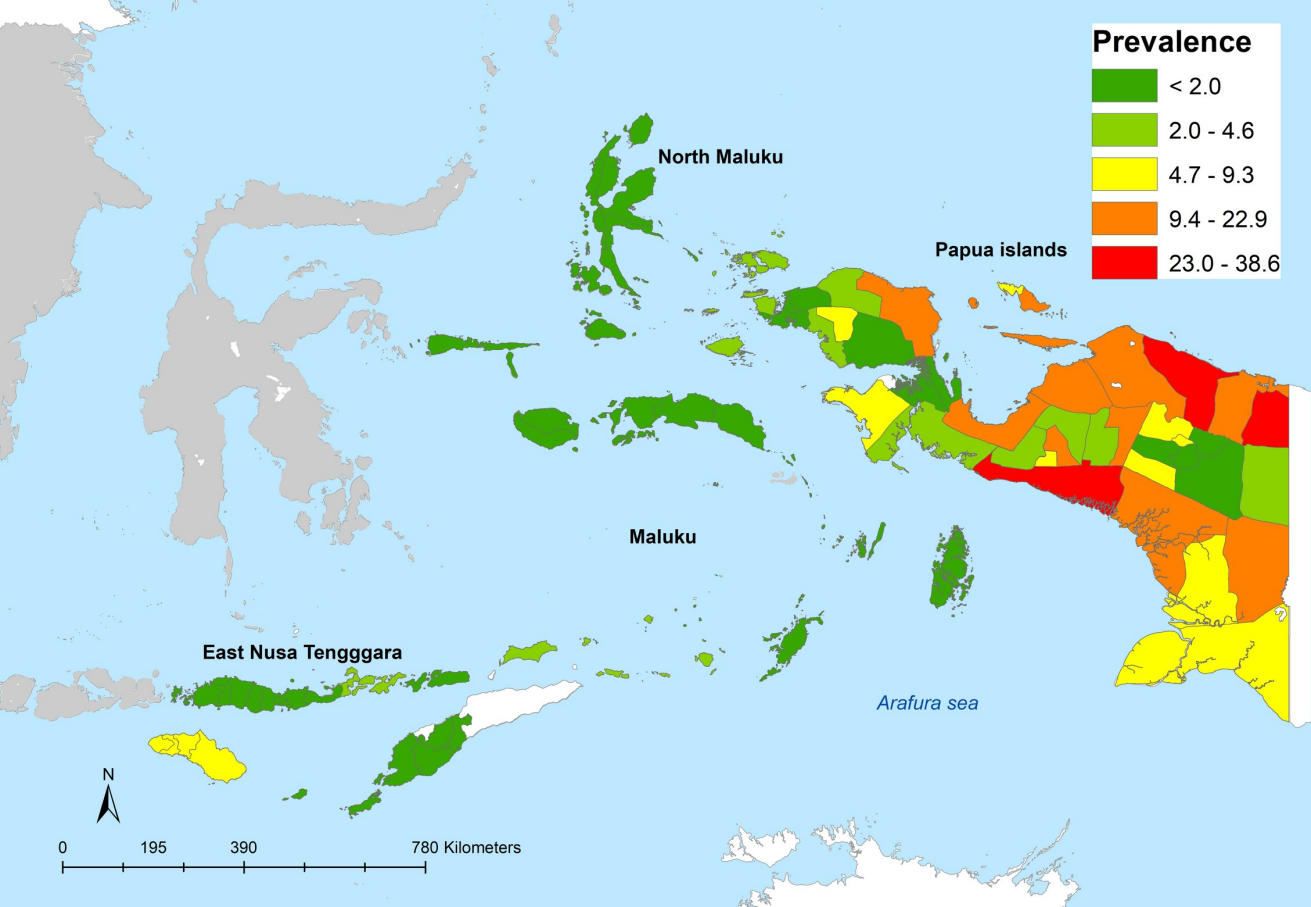
Geographical distribution of self-reported malaria among adults > 15 years old in eastern Indonesia (Riskesdas 2018).

Fig 1 shows the distribution of self-reported malaria prevalence in five provinces studied in eastern Indonesia. A high prevalence of self-reported malaria was identified in districts in Papua, with the highest prevalence was found in Mimika (38.6%), followed by Keerom (38.3%) and Sarmi (27.9%).

### Bivariate analysis

The crude association between malaria and demographical and preventive practices was given in Table 2. The bivariate logistic regressions demonstrated that older participants (aged 25 years above) in rural areas in four provinces were more likely to report having malaria, except in Papua. In all locations, women were less likely to report malaria, compared to males. Education was significantly associated with malaria (p-value < 0.05) in all provinces. Those participants who attained primary education were more likely to report malaria relative to those who did not attend school (Maluku: Odd ratio [OR] = 1.98, 95% confidence interval [CI] = 1.38–2.87; Papua: OR = 1.43, 95%CI 1.38–1.47). Based on occupation, those participants worked in non-agricultural activities more likely to report malaria (Papua: OR = 1.19, 95%CI 1.17–1.21) compared to those who unemployed. This evidence was contradicted with the evidence observed in other sites. Farmers in Maluku, East Nusa Tenggara, West Papua, and Papua were less likely to report malaria relative to those unemployed (Maluku: OR = 0.181, 95%CI 0.143–0.230; East Nusa Tenggara: OR = 0.951, 95%CI 0.924–0.979; West Papua: OR = 0.685, 95%CI 0.656–0.716; Papua: OR = 0.891, 95%CI 0.874–0.908).

**Table 2:** Bivariate analysis of individual and household-level preventive practices and self-reported malaria among adults living in rural areas in five selected provinces in Indonesia.

Variable	Self-reported malaria																			
	Maluku				North Maluku				East Nusa Tenggara				West Papua				Papua			
	Yes	No	P-value	OR (95% CI)	Yes	No	P-value	OR (95% CI)	Yes	No	P-value	OR (95% CI)	Yes	No	P-value	OR (95% CI)	Yes	No	P-value	OR (95% CI)
**Age**																				
15–24 (Ref.)	16	1815			9	1574			112	4669			45	1213			238	2043		
25 and above	53	6160	0.000	11.605 (9.805–13735)	60	5713	0.000	7.635 (6.118–9.528)	415	18058	0.000	1.200 (1.168–1.233)	205	4296	0.000	1.373 (1.317–1.431)	1108	8357	0.221	1.011(0.994–1.028)
**Gender**																				
Male (Ref.)	45	3728			41	3468			248	10671			131	2698			737	5206		
Female	24	4247	0.085	0.854 (0.747–0.976)	28	3819	0.000	0.463 (0.400–0.535)	279	12056	0.000	0.885 (0.867–0.903)	119	2811	0.000	0.836 (0.808–0.865)	609	5194	0.000	0.762 (0.753–0.772)
**Education**																				
No education (Ref.)	3	1235			14	1359			169	6731			52	1230			464	4517		
Primary	18	2543	0.000	1.988 (1.378–2.870)	28	2140	0.000	0.072 (0.053–0.098)	160	7759	0.000	0.983(0.935–1.034)	61	1247	0.000	0.390 (0.365–0.417)	285	2003	0.000	1.432(1.389–1.477)
Secondary	41	3655	0.000	0.254 (0.178–0.362)	24	3228	0.000	0.040 (0.030–0.054)	172	6934	0.000	1.007(0.959–1.058)	114	2444	0.000	0.292 (0.275–0.310)	516	3440	0.000	1.172(1.135–1.209)
Tertiary	7	560	0.020	0.478 (0.340–0.671)	3	560	0.000	0.208 (0.155–0.278)	26	1303	0.000	0.885(0.844–0.928)	23	588	0.000	0.520 (0.492–0.549)	81	440	0.000	1.193(1.159–1.228)
**Occupation**																				
Not working (Ref.)	22	2851			12	2205			148	6162			82	2129			381	3090		
Farmer	33	3555	0.000	0.181 (0.143–0.230)	15	1942	0.000	0.978 (0.803–1.192)	301	12650	0.001	0.951(0.924–0.979)	83	1718	0.000	0.685(0.656–0.716)	691	5787	0.000	0.891(0.874–0.908)
Non-farmer	14	1569	0.000	0.142 (0.119–0.170)	42	3140	0.000	0.403(0.335–0.485)	78	3915	0.001	1.007(0.980–1.035)	85	1662	0.000	0.839(0.802–0.878)	274	1523	0.000	1.192(1.172–1.212)
**Individual preventive measure**																				
Used mosquito nets while slept last night																				
No (Ref.)	57	6828			57	6075			471	18387			201	4590			1045	7679		
Yes	12	1147	0.000	1.943 (1.679–2.250)	12	1212	0.000	0.148 (0.113–0.194)	56	4340	0.000	1.755 (1.701–1.811)	49	919	0.023	1.049 (1.007–1.093)	301	2721	0.000	0.909 (0.896–0.922)
Used insecticide-treated mosquito nets ≤ 3 years																				
No (Ref.)	32	3806			36	4090			140	8272			98	2281			644	5920		
Yes	37	4026	0.000	1.405 (1.239–1.594)	33	3197	0.000	2.627 (2.183–3.163)	387	14455	0.000	0.743 (0.725–0.762)	152	3228	0.000	1.193 (1.156–1.231)	702	4480	0.000	0.699 (0.690–0.707)
Used insecticide-treated mosquito nets > 3 years																				
No (Ref.)	56	6706			56	6257			411	18118			38	1055			279	1893		
Yes	13	13/1282	NA	0.954 (0.802–1.134)	13	1030	0.000	6.305 (5.140–7.735)	116	4609	0.012	0.964 (0.963–0.992)	212	4454	0.000	2.619 (2.467–2.781)	1067	8507	0.000	0.721 (0.709–0.733)
Used mosquito repellent																				
No (Ref.)	57	6772			54	4917			504	20992			189	4341			1084	9107		
Yes	12	1203	0.000	0.376 (0.313–0.451)	15	2370	0.000	2.660 (2.205–3.209)	23	1735	0.000	1.405(1.345–1.468)	61	1168	0.136	0.972(0.937–1.009)	262	1293	0.000	0.663(0.651–0.675)
Used mosquito electric rackets																				
No (Ref.)	67	7781			64	6967			520	22140			240	4991			1304	10171		
Yes	2	194	NA	0.000 (0.000–0.0)	5	320	0.002	1.522 (1.166–1.986)	7	587	0.000	0.865(0.811–0.924)	10	277	0.000	1.373(1.289–1.462)	42	229	0.000	0.927(0.895–0.961)
**Household-level preventive measure**																				
Used mosquito coil/electric anti–mosquito mats																				
No (Ref.)	45	5475			27	3762			476	19422			168	3394			912	7949		
Yes	24	2500	0.000	0.656 (0.568–0.759)	42	3525	0.000	0.564 (0.488–0.651)	51	3305	0.000	1.139 (1.104–1.176)	82	1947	0.298	1.019(0.984–1.054)	434	2451	0.000	0.945(0.930–0.960)
Installed anti-mosquito window screen																				
No (Ref.)	66	7604			67	7046			524	22055			204	4668			1097	9192		
Yes	3	371	0.000	4.507 (3.222–6.303)	2	310	0.000	0.151(0.084–0.272)	3	672	0.000	5.985(5.236–6.841)	46	841	0.000	0.514(0.496–0.532)	249	1208	0.000	0.609(0.598–0.619)

Participants who used bed net while they slept last night (North Maluku: OR = 0.148, 95%CI 0.113–0.194; Papua: OR = 0.909, 95%CI 0.896–0.922). Participants who used ITNs less than three years (East Nusa Tenggara: OR = 0.743, 95%CI 0.725–0.762; Papua: OR = 0.699, 95%CI 0.690–0.707), used ITNs more than three years (East Nusa Tenggara: OR = 0.964, 95%CI 0.963–0.992; Papua: OR = 0.721, 95%CI 0.709–0.733) and used electric anti-mosquito racket (East Nusa Tenggara: OR = 0.865, 95%CI 0.811–0.924; Papua: OR = 0.927, 95%CI 0.895–0.961) were less likely to report malaria. In Maluku and Papua, the study demonstrated that those participants who used repellent were less likely to report malaria (OR = 0.376, 95%CI 0.313–0.451 and OR = 0.663, 95%CI 0.651–0.675, respectively).

At household-level, preventive practices such as using a coil or electric mats likely reduced the odds of reporting malaria among those participants in rural Maluku (OR = 0.656, 95%CI 0.568–0.759), North Maluku (OR = 0.564, 95%CI 0.488–0.651) and Papua (OR = 0.945, 95%CI 0.930–0.960). Applying mosquito screen reduced the likelihood of reporting malaria among participants in North Maluku (OR = 0.151, 95%CI 0.084–0.272), West Papua (OR = 0.514, 95%CI 0.496–0.532) and Papua (OR = 0.609, 95%CI 0.598–0.619).

### Multivariate analysis

The adjusted association between demographical and preventive practices and malaria was indicated in Table 3. By age, older participants (≥25 years) were significantly had higher odds of reporting malaria in all provinces (p<0.001). Females in all provinces were less likely to report malaria, except in Maluku, which indicated that malaria was not statistically significantly correlated with gender (p = 0.085). In Maluku, the odd of reporting malaria were highest in participants who attained secondary-level education (aOR = 1.992, 95%CI 1.779–2.232, p<0.001). Whereas, in Papua, participants who had achieved primary school were more likely to report malaria (aOR = 1.356, 95%CI 1.039–1.768). Participants in North Maluku, East Nusa Tenggara, and West Papua, those who well-educated were less likely to report malaria (p<0.001). Those participants who worked as a farmer were more likely to report malaria (Maluku, aOR = 1.490, 95%CI 1.377–1.612, p<0.001) but not in North Maluku (aOR = 0.725, 95%CI 0.677–0.777, p<0.001) and West Papua (aOR = 0.805, 95%CI 0.775–0.837, p<0.001).

**Table 3:** Association between individual and household-level preventive practices and self-reported malaria among adults living in rural areas in five selected provinces in Indonesia.

Variable	Self-reported malaria																			
	Maluku				North Maluku				East Nusa Tenggara				West Papua				Papua			
	Yes	No	P-value	aOR (95% CI)	Yes	No	P-value	aOR (95% CI)	Yes	No	P-value	aOR (95% CI)	Yes	No	P-value	aOR (95% CI)	Yes	No	P-value	aOR (95% CI)
**Age**																				
15–24 (Ref.)	16	1815			9	1574			112	4669			45	1213			238	2043		
25 and above	53	6160	0.000	2.504 (2.310–2.715)	60	5713	0.000	1.917 (1740–2.112)	415	18058	0.000	1.065 (1.039–1.091)	205	4296	0.000	1.580 (1.521–1.641)	1108	8357	NA	NA
**Gender**																				
Male (Ref.)	45	3728			41	3468			248	10671			131	2698			737	5206		
Female	24	4247	0.020	0.554 (0.522–0.587)	28	3819	0.000	0.628 (0.591–0.668)	279	12056	0.000	0.949 (0.932–0.967)	119	2811	0.000	0.949 (0.921–0.978)	609	5194	0.002	0.833 (0.742–0.935)
**Education**																				
No education (Ref.)	3	1235			14	1359			169	6731			52	1230			464	4517		
Primary	18	2543	0.000	1.590 (1.407–1.796)	28	2140	0.000	0.200 (0.165–0.243)	160	7759	0.002	0.927 (0.884–0.972)	61	1247	0.000	0.726 (0.685–0.769)	285	2003	0.025	1.356 (1.039–1.768)
Secondary	41	3655	0.000	1.992 (1.779–2.232)	24	3228	0.000	0.164 (0.136–0.197)	172	6934	0.325	0.977 (0.932–1.024)	114	2444	0.000	0.521 (0.494–0.549)	516	3440	0.610	1.074 (0.816–1.414)
Tertiary	7	560	0.000	1.126 (1.019–1.245)	3	560	0.000	0.343 (0.285–0.412)	26	1303	0.000	0.797 (0.762–0.834)	23	588	0.000	0.698 (0.666–0.731)	81	440	0.629	1.066 (0.823–1.381)
**Occupation**																				
Not working (Ref.)	22	2851			12	2205			148	6162			82	2129		Ref	381	3090		
Farmer	33	3555	0.000	1.490 (1.377–1.612)	15	1942	0.000	0.725 (0.677–0.777)	301	12650	0.881	1.002 (0.976–1.029)	83	1718	0.000	0.805 (0.775–0.837)	691	5787	NA	NA
Non-farmer	14	1569	0.000	0.750 (0.698–0.805)	42	3140	0.000	1.229 (1.123–1.346)	78	3915	0.000	1.105 (1.078–1.133)	85	1662	0.000	0.888 (0.853–0.925)	274	1523	NA	NA
**Individual preventive measure**																				
Used mosquito nets while slept last night																				
No (Ref.)	57	6828			57	6075			471	18387			201	4590			1045	7679		
Yes	12	1147	0.000	0.560 (0.525–0.596)	12	1212	0.000	1.282 (1.181–1.393)	56	4340	0.000	1.546 (1.504–1.590)	49	919	NA	NA	301	2721	0.053	1.147 (0.998–1.318)
Used insecticide-treated mosquito nets ≤ 3 years																				
No (Ref.)	32	3806			36	4090			140	8272			98	2281			644	5920		
Yes	37	4026	0.000	0.664 (0.629–0.700)	33	3197	0.000	1.364 (1.281–1.452)	387	14455	0.000	0.673(0.658–0.689)	152	3228	0.000	1.190(1.157–1.224)	702	4480	0.000	0.695(0.618–0.782)
Used insecticide-treated mosquito nets > 3 years																				
No (Ref.)	56	6706			56	6257			411	18118			38	1055			279	1893		
Yes	13	13/1282	0.592	NA	13	1030	0.000	0.690 (0.642–0.741)	116	4609	0.000	0.869 (0.847–0.893)	212	4454	0.000	2.842 (2.689–3.003)	1067	8507	0.019	0.839 (0.725–0.972)
Used mosquito repellent																				
No (Ref.)	57	6772			54	4917			504	20992			189	4341			1084	9107		
Yes	12	1203	0.000	0.633 (0.592–0.676)	15	2370	0.000	2.229 (2.075–2.396)	23	1735	0.000	1.256 (1.207–1.307)	61	1168	0.000	0.937 (0.909–0.967)	262	1293	0.000	0.738 (0.626–0.874)
Used mosquito electric rackets																				
No (Ref.)	67	7781			64	6967			520	22140			240	4991			1304	10171		
Yes	2	194	0.995	NA	5	320	NA	NA	7	587	0.013	0.925 (0.870–0.983)	10	277	0.000	1.537 (1.450–1.630)	42	229	NA	NA
**Household-level preventive measure**																				
Used mosquito coil/electric anti–mosquito mats																				
No (Ref.)	45	5475			27	3762			476	19422			168	3394			912	7949		
Yes	24	2500	0.000	0.838 (0.792–0.886)	42	3525	0.000	0.479 (0.451–0.509)	51	3305	0.000	1.202 (1.158–1.238)	82	1947	NA	NA	434	2451	0.015	0.838 (0.726–0.967)
Installed anti-mosquito window screen																				
No (Ref.)	66	7604			67	7046			524	22055			204	4668			1097	9192		
Yes	3	371	0.000	NA	2	310	0.000	2.291 (1.851–2.835)	3	672	0.000	7.082 (6.196–8.094)	46	841	0.000	0.548 (0.531–0.565)	249	1208	0.000	0.701 (0.597–0.824)

At the individual level, using a bed net while slept last night, have used ITNs less than five years and using repellent likely reduced the odd of reporting malaria among Maluku participants by 44% (95%CI 41–48%), 34% (30–37%) and 37% (32–41%), respectively. In North Maluku, the odd of reporting malaria were lower among participants who used ITNs for more than three years (aOR = 0.690, 95%CI 0.642–0.741). Participants in rural East Nusa Tenggara who used ITNs (aOR = 0.673, 95%CI 0.658–0.689) and electric anti-mosquito racket (aOR = 0.925, 95%CI 0.870–0.983) were less likely to report malaria. In West Papua, only participants who used repellent were less likely to report malaria (aOR = 0.937, 95%CI 0.909–0.967). In Papuan rural communities, the odds of reporting malaria were lower in those who used ITNs (aOR = 0.695, 95%CI 0.618–0.782) and repellent (aOR = 0.738, 95%CI 0.626–0.874).

At the household-level, using the burnt coil/electric anti-mosquito mats reduced the odds of reporting malaria by 17% (12–21%) (Maluku), 52% (50–65%) (North Maluku) and 16% (3–27%) (Papua). The odds of reporting malaria were lower in that rural communities in West Papua (aOR = 0.548, 95%CI 0.531–0.565) and Papua (aOR = 0.701, 95%CI 0.597–0.824) who installed mosquito window screen in their house.

## Discussion

Our study analyzed the subset of data collected from recent Indonesia’s national-representative community-based survey (RISKESDAS 2018). RISKESDAS aimed to describe preventive practices applied by rural communities in five major endemic areas in eastern Indonesia and to examine the association between self-reported malaria among adults population aged more than 15 years old and demographical and identified preventive practices. In general, while there was a variation between location, our study reveals that age, gender, occupation, and education were associated with self-reported malaria. The study demonstrates that there is a considerable variation of approaches in preventing the bite of malaria vectors among rural communities living in areas studied.

In this study, we identified that males and older participants (aged 25 years above) were more likely to report malaria in rural areas in four provinces, except in Papua. Our finding is consistent with the study in Sub-Saharan and South Africa. It was reported that malaria is a significant public health issue among adults and more pronounced in the economically active male populations [33,34]. For instance, a study in Kenya showed *Plasmodium falciparum* infection was associated with male, poor, and malnourished infection [34]. Women were less likely to be reported in malaria compared to males. These findings were similar to studies elsewhere [19,35,36] and a previous study in Indonesia [37].

Further, the present study also revealed intriguing evidence on the association between self-reported malaria and occupation. In Maluku, North Maluku, ENT, and West Papua, farmers were less likely to report malaria relative to those who unemployed. While in Papua, the higher odd of self-reporting malaria were observed among people who worked in the non-agricultural sector (Papua). These findings are inconsistent with that previous study, which also conducted in the same location [33]. The difference in the statistical approach used by both studies might partly explain this contradictory finding. This study also showed that education was associated with self-reported malaria. Our research suggests that people having lower education background was more likely to report malaria. Both occupations and education are important risk factors related to malaria, and it strongly reflects an individual’s socioeconomic status. This disparity could be explained by the fact that people with better education would usually be more well-informed and aware of malaria. Therefore they were more likely to either over-report malaria (Papua) or be aware of malaria prevention (East Nusa Tenggara) activities.

Our study demonstrates that using a bed net while slept likely reduced the odd of malaria in Maluku, but no in the other two sites (North Maluku and East Nusa Tenggara). The use of ITNs also seemed to be less protective in some areas, although the use of ITN is considered as one of the most cost-effective and preventive malaria strategies. These results are consistent with several studies finding that preference to unused the insecticide-treated bed nets (ITNs) projected an increased incidence of malaria [38–42]. The heterogeneity in the effects of bed-net use and malaria incidence has been indicated in southern Ethiopia, in which the prevalence of malaria was also high despite the frequent use of bed-net [43]. A study carried out in Malawi aimed to evaluate the effectiveness of ITNs in preventing malaria reveals that there was no significant personal protective effect of ITNs found [44].

In contrast, a study conducted in Southwestern Nigeria showed that the use of ITN, along with educational programs, could dramatically reduce the prevalence of *Plasmodium falciparum* malaria parasitemia by 5% at three months [45]. The variabilities partly explained by the compliance level variation among individuals and also the efficacy of the existing ITNs in the community. This case causes an insignificant protective effect of ITN because the use of ITN does not fit its intended purpose [44]. ITNs have become an essential tool in malaria control. Therefore there is a need to routinely monitor and evaluate the utilization of ITNs and its efficacy.

This study was reported, those participants who used repellent in Maluku, West Papua and Papua were less likely to report malaria. Similar with clinic study in Chennai and Raurkela, India found a correlation between repellents and malaria [46] This is in contrast with findings in Afghanistan which showed no significant reduction in malaria infections in adults over the age of 20 [47]. Similar evidence in Cambodia also reported there were although entomological data show that the Picaridin repellent reduces 97% of mosquito bites in over five hours, nevertheless there could be no reduction in malaria prevalence [48]. Other study findings, which aims to assess the impact of topical repellents, insecticide-treated clothing, and spatial repellents on malaria transmission, reported that there is not enough evidence to conclude that repellents from local or spatial sources can prevent malaria [49]. Several contrast findings with this result study could be explained that there was a strong preference for frequent application of repellent. It was noted when performing economic and subsistence activities in the forest, mostly by men, and in places where insect nuisance is high. As insect nuisance is one of the central stimulants for repellent use also in other contexts [50–52]

Our study demonstrates that coils or applied electric anti-mosquito mats and installed mosquito window screen could reduce the odds of reporting malaria. Still, it seemed less protective among participants in East Nusa Tenggara. Plausible explanation regarding prevention practices at the household level might be because they only protected individuals inside the house. Meanwhile, the risk of malaria transmission likewise occurs outside of the house. Considering the risk of malaria mosquito vector biting is primarily outdoor [53,54], household prevention measures may not be very effective [55]. Our study found that, comparatively, in all provinces, most of the households used coils than the installed window screen. This finding consistent with the results of a study in Nigeria. It was reported that malaria infection was higher among people who stayed in unimproved houses [56]. Another study carried out in Uganda showed that in modern homes, the human biting rate was lower compared to traditional homes. In all sub-counties, the risks of malaria infection for modern homes are smaller [57]. Houses are not the only place where malaria is transmitted, but they are still the most crucial transmission environment in many endemic areas [58,59]. House improvements refer to the complete screening or closure of openings such as windows, doors, and eaves or ceiling construction. The aim is to reduce indoor interaction between mosquitoes and humans. Although biting and transmission happening in outdoors, there is evidence that mosquito is likely to enter a house at some point during their lifetime before an infective bite is delivered. [60] A study in Gambian where 500 households were assigned in a village with full screening, screened ceilings, or no screening at all showed that more mosquitoes were trapped in houses that had no screening compared to the rest of the houses [61].

While the study provides some significant findings, it also has some limitations. First, this study is cross-sectional so that we could not be able to infer the causal relationships between malaria and preventive practices. Second, it should be noted that the outcome variable of this study was self-reported malaria. No diagnostic tests were performed to confirm malaria infection among participants. Consequently, the findings should be carefully interpreted as this could subject to biases (e.g., reporting bias and/or social desirability bias).

## Conclusions

In conclusion, this study demonstrated substantial variation in the effects of preventive practices on malaria infection among adult populations in rural communities among localities over endemic areas in eastern Indonesia. The results of this study suggest the need for strengthening Information, Education and Communication strategies along with current intervention efforts. In particular, monitoring and evaluation on the utilization of ITNs in the communities should be routinely implemented.
